# Brain Transcriptional Responses to High-Fat Diet in *Acads*-Deficient Mice Reveal Energy Sensing Pathways

**DOI:** 10.1371/journal.pone.0041709

**Published:** 2012-08-22

**Authors:** Claudia Kruger, K. Ganesh Kumar, Randall L. Mynatt, Julia Volaufova, Brenda K. Richards

**Affiliations:** 1 Pennington Biomedical Research Center, Louisiana State University System, Baton Rouge, Louisiana, United States of America; 2 School of Public Health, Louisiana State University Health Sciences Center, New Orleans, Louisiana, United States of America; Karlsruhe Institute of Technology, Germany

## Abstract

**Background:**

How signals from fatty acid metabolism are translated into changes in food intake remains unclear. Previously we reported that mice with a genetic inactivation of *Acads* (acyl-coenzyme A dehydrogenase, short-chain), the enzyme responsible for mitochondrial beta-oxidation of C4–C6 short-chain fatty acids (SCFAs), shift consumption away from fat and toward carbohydrate when offered a choice between diets. In the current study, we sought to indentify candidate genes and pathways underlying the effects of SCFA oxidation deficiency on food intake in *Acads−/−* mice.

**Methodology/Principal Findings:**

We performed a transcriptional analysis of gene expression in brain tissue of *Acads−/−* and *Acads+/+* mice fed either a high-fat (HF) or low-fat (LF) diet for 2 d. Ingenuity Pathway Analysis revealed three top-scoring pathways significantly modified by genotype or diet: oxidative phosphorylation, mitochondrial dysfunction, and CREB signaling in neurons. A comparison of statistically significant responses in HF *Acads−/−* vs. HF *Acads+/+* (3917) and *Acads+/+* HF vs. LF *Acads+/+* (3879) revealed 2551 genes or approximately 65% in common between the two experimental comparisons. All but one of these genes were expressed in opposite direction with similar magnitude, demonstrating that HF-fed *Acads*-deficient mice display transcriptional responses that strongly resemble those of *Acads+/+* mice fed LF diet. Intriguingly, genes involved in both AMP-kinase regulation and the neural control of food intake followed this pattern. Quantitative RT-PCR in hypothalamus confirmed the dysregulation of genes in these pathways. Western blotting showed an increase in hypothalamic AMP-kinase in *Acads−/−* mice and HF diet increased, a key protein in an energy-sensing cascade that responds to depletion of ATP.

**Conclusions:**

Our results suggest that the decreased beta-oxidation of short-chain fatty acids in *Acads*-deficient mice fed HF diet produces a state of energy deficiency in the brain and that AMP-kinase may be the cellular energy-sensing mechanism linking fatty acid oxidation to feeding behavior in this model.

## Introduction

Fatty acid oxidation is implicated in the metabolic control of food intake and energy balance [1]. However, the signal(s) mediating the effects of fatty acid oxidation on food intake has not yet been identified [2]–[3]. A number of studies indicate that enzymes within fatty acid metabolic pathways may serve as targets for pharmacological tools to treat obesity [4], yet there is controversy over which aspect of fatty acid availability may inhibit food intake. We developed a novel approach for investigating the molecular and physiological links between fat intake and fat oxidation using an animal model with a genetic deficiency in short-chain acyl-CoA dehydrogenase (*Acads*), a mitochondrial enzyme that catalyzes the first reaction in the beta-oxidation of C4-C6 fatty acids. A spontaneous deletion in the structural gene for *Acads* results in the complete absence of functional short-chain acyl-CoA dehydrogenase [5] in the BALB/cByJ mouse inbred strain. We have shown that *Acads*-deficiency [6]–[7] suppressed voluntary fat- but not carbohydrate intake in mice [8]. Specifically, *Acads*−/− mice shifted their food consumption away from fat and toward carbohydrate when offered a choice of diets, while total calorie intake remained unchanged. In addition we showed that during brief access lick tests, *Acads*−/− mice showed similar licking responses to fat and carbohydrate stimuli compared with *Acads*+/+ controls suggesting that their fat avoidance is not mediated by an orosensory mechanism. In the current study, we tested the hypothesis that gene expression patterns in *Acads*-deficient mice would reveal functionally important pathways mediating the behavioral feeding response to fat.

Energy balance is maintained by changes in behavior and metabolism which can be regulated by gene expression. To our knowledge, there are no reports of studies on the effects of dietary fat level on the brain transcriptome in genetic models of fatty acid oxidation deficiency. Microarray analysis of the effects of *Acads* deficiency on gene expression in brain is limited to a single investigation in which animals were fed standard chow and the number of genes/ESTs represented on the array (<5,000) was relatively small [9]. The purpose of the current study was to identify key transcriptional processes linked to fat intake, and ultimately to behavior, in a genetic model of impaired SCFA oxidation. We used microarray technology to compare the short-term effects of high- or low-fat diet on gene expression in the brain of *Acads*−/− and *Acads+/+* mice. Tissues were harvested 2 d after initiating the experimental diets to coincide with the time-point at which fat avoidance begins [8]. We identified transcriptional responses to genotype and diet that that may be linked to feeding behavior in this model. We also investigated gene expression in hypothalamus because of its role in the regulation of metabolic and behavioral phenotypes.

## Materials and Methods

### Experimental animals

The BALB/cByJ (*Acads−/−*) and BALB/cByKZ (*Acads+/+*) mice were obtained from breeding colonies maintained at the Pennington Biomedical Research Center (PBRC). The BALB/cByJ mouse strain carries a spontaneous 278 bp deletion in the structural gene for *Acads* that occurred spontaneously sometime between 1981 and 1982 [5]–[7], [10]–[12]. The BALB/cByJ mice are descendents of the BALB/cBy strain maintained originally by Donald Bailey at the Jackson Laboratory. The best *Acads+/+* control line for BALB/cByJ mice is thought to be the BALB/cBy [13]. The BALB/cByKZ.*Acads+/+* substrain was separated from the research colonies at the Jackson Laboratory in 1996. Due to the length of time that these substrains have been separated, we cannot rule out undetected spontaneous mutations in genes other than *Acads* that could affect gene expression. All protocols were approved by the Institutional Animal Care and Use Committee of Pennington Biomedical Research Center.

### Experimental protocol

Twelve-week old male mice were singly housed in filter-top cages and kept under 12 h light/12 h dark conditions at an ambient temperature of 22–23°C in a specific-pathogen free facility. Several days prior to the experiment, bedding was removed and replaced with stainless steel wire floor inserts, to permit recovery and measurement of food spillage. Polyvinylchloride nesting tubes (1½ in. diameter) were provided to reduce time spent on wire flooring. The high- (HF) and low-fat (LF) experimental diets were equivalent for protein (16.4% of energy) with the balance of calories contributed by 58% fat and 25.5% carbohydrate in D12331 and by 10.5% fat and 73.1% carbohydrate in D12329 (Research Diets, Inc., New Brunswick, NJ) (Table S1).

Gene expression profiles were compared in whole brain using a diet x genotype design. The body weights of *Acads−/−* (28.8±0.7 g) and *Acads+/+* (29.2±0.4 g) mice were similar at baseline. Daily food intake was monitored during the 2 d experiment and there were no significant differences in total calorie intake between genotype or diet groups: HF *Acads−/−* (27±1) vs. HF *Acads+/+* (27±1), *P = *0.90; LF *Acads−/−* (26±1) vs. LF *Acads+/+* (25±2), *P* = 0.67; LF HF *Acads+/+* (27±1) vs LF *Acads+/+* (25±2), *P* = 0.33; HF *Acads−/−* (27±1) vs LF *Acads−/−* (26±1), *P* = 0.37. Three mutant (*Acads−*/−) and three wild-type (*Acads+/+*) mice of each diet group were euthanized with CO_2_ between 10:00 and 11:00 AM in the morning of dissection. Brains were quickly removed, frozen in liquid nitrogen and stored at −80°C. Twelve individual RNA samples (3 per strain and diet) were labeled and hybridized according to the manufacturer's protocol. Thus, three biological replicates were analyzed for each strain and condition.

### Microarray analysis

Total RNA was isolated from whole brain using TRI Reagent (Molecular Research Center, Inc., Cincinnati, OH, USA), and the quality was determined using an Agilent Bioanalyzer 2100 (Agilent Technologies, Santa Clara, CA, USA). One µg of total RNA was used to transcribe DIG-labeled cRNA using AB Chemiluminescent RT-IVT Kit v2.0. Microarray hybridization (using 10 µg of fragmented, DIG-labeled cRNA), processing, chemiluminescence detection, imaging, auto-gridding, and image analysis were performed according to AB protocols, using the 1700 Chemiluminescent Microarray Analyzer Software v. 1.0.3. The Applied Biosystems Mouse Genome Survey Microarray V2.0 was used which contains ∼34,000 features, including a set of ∼1000 controls. Each microarray uses 32,996 probes targeted to 32,381 curated genes representing 44,498 transcripts. The AB Expression system software was used to extract assay signal and signal-to-noise ratio values from the images. Signal intensities across microarrays were normalized according to the quantile-quantile method based on R-script [14]; http://www.bioconductor.org). Features with a signal/noise value of ≥3.0 and quality flag value of <5000 were considered “detected” and subjected to statistical analysis using AB array. The FDR-corrected t-statistic was calculated using the Benjamini-Hochberg procedure [15]. Differential expression was defined as a fold change ≥1.6 and a *P* value of ≤0.05. The microarray data, described according to MIAME guidelines, have been deposited in the National Center for Biotechnology Information (NCBI) Gene Expression Omnibus (GEO) repository. The accession number is GSE35180.

### Acylcarnitine analyses

Acylcarnitines were measured by the Analytical Systems Laboratory of the Louisiana State University School of Veterinary Medicine. Plasma samples were analyzed by direct-injection electrospray tandem mass spectrometry, using a Quattro II LC-MS system (Waters-Micromass, Milford MA) equipped with an HTS-PAL autosampler (Leap Technologies, Carrboro, NC). An Agilent 1100 HPLC system with a binary pump (Agilent Technologies, Santa Clara, CA) was interfaced to the mass spectrometer. All data were acquired and analyzed using MassLynx software, version 4.0 (Waters-Micromass).

### Ingenuity Pathway analysis (IPA)

Differentially expressed genes (>1.6-fold) with a comparison *P* value of <0.05 were subjected to Ingenuity Pathways Analysis (www.ingenuity.com) (Ingenuity Systems, Mountain View, CA). The IPA is a manually curated database of molecular interactions and contains previously published findings from peer-reviewed publications. It should be noted that the interactions presented in the networks are not specific for brain tissue. Fischer's exact test was used to calculate *P* values determining the probability that the association between the genes in the dataset and pathway could be explained by chance alone. The significance score was expressed as the negative logarithm of the *P* value, indicating the likelihood that the focus genes were assembled into a pathway.

### Real-time quantitative PCR

*cDNA preparation for RT-PCR*. RNA quality was assessed using the Agilent 2100 bioanalyzer (Agilent Technologies, Santa Clara, CA, USA), and quantity was determined in the NanoDrop ND-1000 Spectrophotometer (NanoDrop Technologies, Rockland, DE, USA). Complementary cDNA was obtained by reverse transcription (SuperScript III First-Strand Synthesis System for RT-PCR, Invitrogen, Carlsbad, CA, USA) of 4 µg of RNA from each sample. This reaction used both Oligo(dT)_20_ and Random Hexamers as primers; all further steps were done following the supplier's instructions (Invitrogen, Carlsbad, CA, USA). Purification of cDNA was performed using QIAquick PCR purification columns (Qiagen, Valencia, CA, USA). For quantitative RT-PCR, 6–12 samples each were tested for the *Acads−/−* and *Acads+/+* strains using a combination of the microarray samples (3 per strain and condition) and others.

Primers for amplification were designed using Primer Express 3.0 software from Applied Biosystems (AB; Foster City, CA, USA) and synthesized by IDT (Integrated DNA Technologies, Coralville, IA, USA). The locations and sequences of primer are listed in Table S2. Primers were designed, when possible, to span an exon/exon junction to avoid amplification from potentially contaminating genomic DNA. Primer sequences were subjected to BLAT analysis (http://genome.ucsc.edu) to confirm their specificity. The ENSEMBLE genome browser contains the transcript and exon information for the genes investigated in this study (Table S2 with accession numbers given).

The MultiPROBE II PLUS HT EX robot (Perkin Elmer, Shelton, CT, USA) was programmed to pipette 10 µl reactions into an AB 384 well plate. The robot adds 3 µl template (2 ng cDNA) and 7 µl Master Mix (5 µl iTaq SYBR Green Supermix with ROX, 0.1 µl forward primer 10 µM, 0.1 µl reverse primer 10 µM, 1.8 µl NanoPure water) per reaction. The iTaq SYBR Green Supermix with ROX (2×) was obtained from Bio-Rad Laboratories (Hercules, CA, USA). Gene expression levels were measured using the ABI PRISM 7900HT Sequence Detection System with SDS 2.3 software version (AB). Individual samples were run in triplicate. As endogenous reference for normalization, we used the measurement of Cyclophilin (Ppia) cDNA in the same sample; *Ppia* levels were unaffected by diet genotype on the arrays.

Relative quantification was carried out using the comparative C_T_ method (AB: User Bulletin #2: Relative quantification of gene expression. P/N 4303859). A two-tailed Student's *t*-test was performed on delta Ct values to evaluate strain differences in gene expression. *P* values of ≤0.05 were considered statistically significant.

### Protein analysis by immunoprecipitation and immunoblotting

Total protein was isolated from hypothalamus using Lysis buffer (25 mM HEPES pH 7.8, 1% NP-40, 50 mM KCl, 125 µM DTT, 1 mM PMSF, 5 mM NaPPi, 1 mM EDTA, 50 mM NaF including protease inhibitor cocktail). Protein concentration was determined with the BCA assay (Pierce Biotechnology, Rockford, IL). Electrophoretic separations were carried out on Mini-Protean II electrophoresis cells (Bio-Rad Laboratories, Inc., Hercules, CA) and run at 100V. 45 µg of proteins were separated on a 10% SDS-PAGE and transferred onto PVDF membranes (Bio-Rad) using a Mini Trans-Blot (Bio-Rad). The membranes were blocked in 5% milk in TBST and then immunoblotted overnight at 4 deg C with rabbit anti-phospho-AMPK alpha1, 2 (Thr^172^) or rabbit anti-AMPK (Abcam Inc., Cambridge, MA). After extensive washing, the membranes were incubated with goat anti-rabbit horseradish peroxidase-linked secondary antibody (Abcam). Membranes were washed again and proteins were visualized using the Pierce enhanced chemi-luminescence Western blotting detection system. Mouse monoclonal antibodies to beta Actin (HRP) (Abcam) were used as loading control. Densitometry of Western blots was performed using Image J Software [16].

### Statistical analyses

Acylcarnitine data were analyzed using an analysis of variance approach (ANOVA) with Tukey's adjustment for pair-wise comparisons. The testing procedure focused on the main effects of diet and strain, as well as the diet by strain interaction. The level for statistical significance was set at *P*<0.05. The SAS version 9.2.1 statistical software package was used.

## Results

The present study was designed to identify the brain transcriptional responses to genotype and diet that may be linked to feeding behavior in this model of *Acads* deficiency. To further clarify the microarray results in total brain (see Text S1), we investigated gene expression in the hypothalamus, because of its role in the regulation of metabolic and behavioral phenotypes.

### Plasma acylcarnitine levels

**Figure 1:**
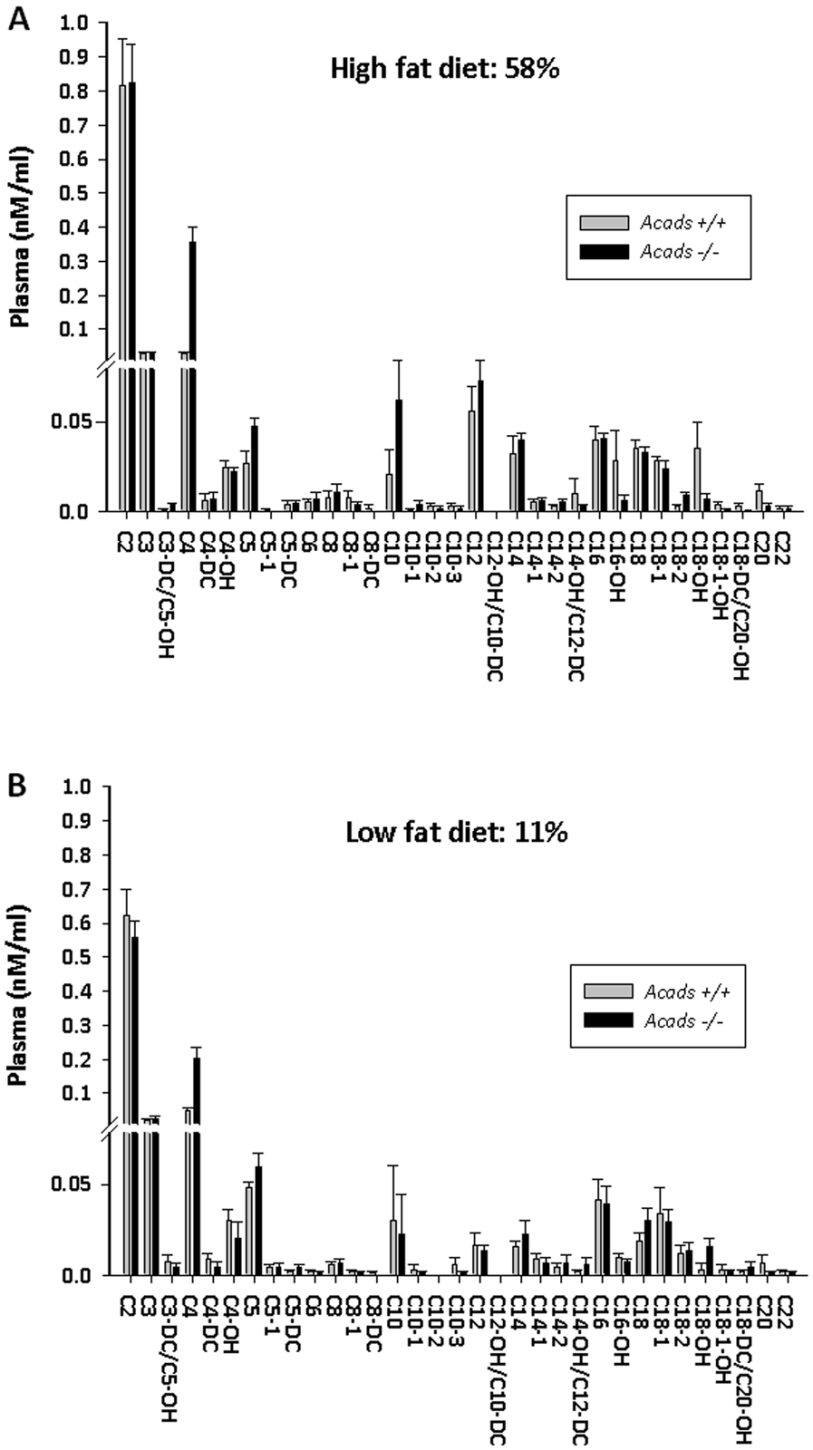
Effects of genotype and diet on plasma acylcarnitine levels. Acylcarnitines were measured by tandem mass-spectrometry in plasma from *Acads−/−* and *Acads+/+* mice fed high- (A) or low-fat (B) diet. Values are means (pmol/mg protein) ± SEM. n = 12 animals per group. **P<0.0001, *P<0.01, #*P*<0.05 for genotype comparisons. See Table S3 for a summary of ANOVA results for these variables.

Tandem mass spectrometry was used to analyze 33 independent acylcarnitine species ranging in size from 2 to 22 carbons, in the plasma of *Acads−/−* and *Acads+/+* mice fed HF (58%) or LF (11%) fat diet for 2 days. Plasma levels of seven acylcarnitines showed a main effect of Diet and three acylcarnitines showed a main effect of Strain (Table S3). Specifically, on HF diet the plasma content of C4 and C5 was 13-fold (*P*<0.0001) and 2-fold (*P*<0.05) higher, respectively, in *Acads−/−* compared with *Acads+/+* mice (Fig. 1). On LF diet, a 2.5-fold increase in circulating C4 (*P*<0.01) was observed in *Acads−/−* compared with *Acads+/+* mice. The substantial increase in plasma acylcarnitine levels in SCAD-deficient mice points to the location of the metabolic block and demonstrates an effect of short-term HF diet. Only two acylcarnitines showed a significant Diet x Strain interaction: C4 [*F*(3,21) = 9.55, *P*<0.01] and C18-OH [*F*(3,21) = 7.6, *P*<0.05]. The physiological significance of the altered C18-OH levels is unknown.

### Gene expression analysis

Expression levels of 32,381 transcripts in the brain were compared between genotypes on HF or LF diet, and between diets for *Acads−/−* or *Acads+/+* respectively. Only transcripts whose expression levels showed a fold change of ≥1.6 with a corresponding *P* value of <0.05 were analyzed. Transcripts were also sorted based on gene ontologies assigned in the MGI database version 3.0 (Fig. S1).

### Effects of high- or low-fat diet on gene expression

**Figure 2:**
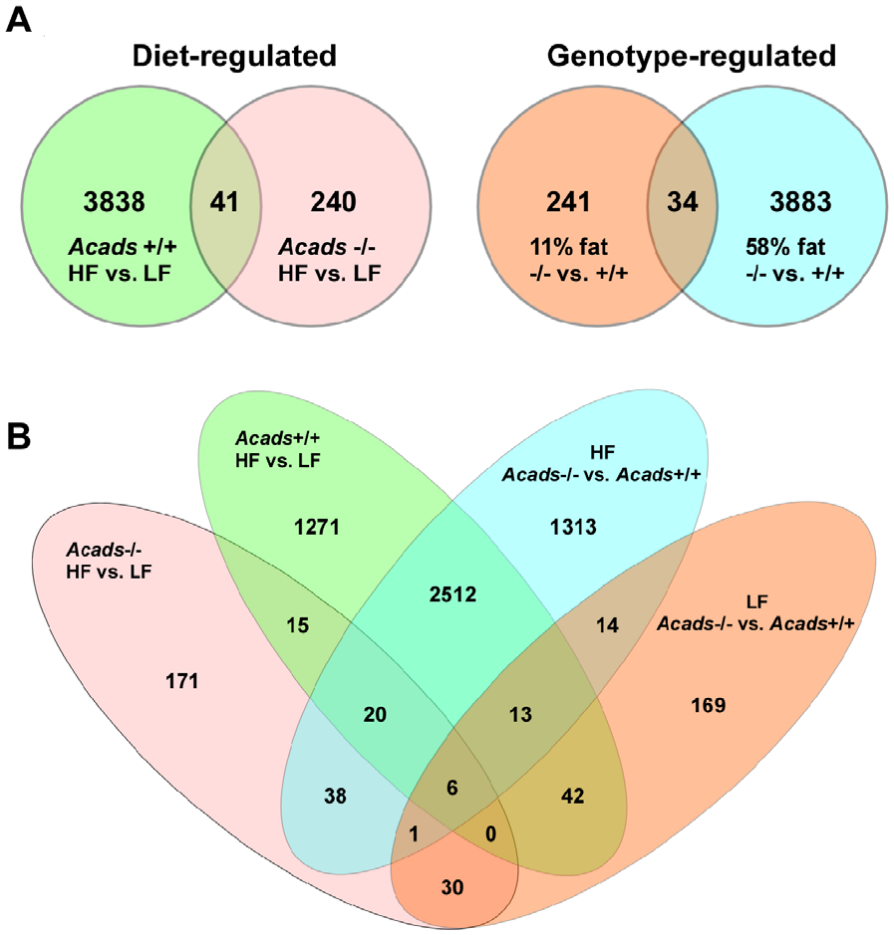
Venn diagrams of gene totals disturbed by genotype and diet. Two-way (A) and four-way (B) Venn diagrams of diet and genotype effects on gene expression in *Acads−/−* and *Acads+/+* mice fed high- or low-fat diet. Only genes with changes >1.6-fold and *P*<0.05 were included. See Table 1 for the total number of differentially expressed genes for each of the four comparisons. Within each main comparison (e.g., separate colors in B), the overlap from sub-categories was subtracted from the total number of diet- or genotype-regulated transcripts. Only six transcripts were found in common among all four comparisons.

Strikingly, the expression of nearly four thousand genes (3879) was significantly deregulated in wildtype controls after 2 d of HF diet feeding, and more than one-half of these genes showed increased expression (Table 1). By contrast, only 281 genes were differentially expressed as an effect of diet in *Acads*-deficient animals and most of those were decreased. These results clearly indicate that the lack of functional SCAD enzyme diminished the animals' ability to respond to HF diet. Forty-one (41) genes were regulated by diet in both *Acads−/−* and *Acads+/+* mice (Figure 2A) and of these, eighteen (18) were differentially expressed in opposite direction. Specifically, we identified diet-regulated genes involved in processes such as regulation of cell matrix adhesion (*Plekha2*), ATP catabolic process (*Atp7a*), protein folding (*Dnajb9*), development (*Pax7*), adrenal gland function (*Nr3c1*), RNA binding (*Oas1c*), and copper ion homeostasis (*Aplp2*). These genes were strongly down-regulated by dietary fat (>2-fold) in *Acads*-deficient animals, but were up-regulated by HFD in controls.

**Table 1:** Summary of differentially expressed genes.

Experimental comparison	Total	Increased	Decreased
HF: Acads−/− vs. Acads+/+	3917	1737	2180
LF: Acads−/− vs. Acads+/+	275	220	55
Acads+/+: HF vs. LF	3879	2156	1723
Acads−/−: HF vs. LF	281	52	229

The Applied Biosystems Mouse Genome Survey Microarray v2.0 was used to identify gene expression changes in whole brain as an effect of genotype and dietary fat level.

A total of 12 arrays were used. Lists of differentially expressed, unique genes were Generated for each comparison based on a fold change of ≥1.6 and a *P* value of <0.05.

### Effects of *Acads* genotype on gene expression

With HF diet, the expression of 3917 genes was altered as an effect of genotype (*Acads−/−* vs *Acads+/+*), compared with 275 genes with LF diet (Table 1). Thirty-four (34) genes were regulated by genotype under both HF and LF diet conditions (Fig. 2A), and of these, sixteen (16) genes were changed in opposite direction. For example, we identified transcripts that were strongly down-regulated in *Acads*-deficient mice and up-regulated in controls under both diet conditions, including genes involved in processes such as AMP-activated protein kinase activity (*Pkig*, *Prkag1*, *Stk38*, *Ccdc88a*), protein folding (*Dnajb9*), cell adhesion (*Pcdhb8*), ATP binding (*Ddx6*), and regulation of transcription (*Carf*).

### Effects of *Acads* genotype and diet on gene expression: 4-way Venn diagram

A four-way Venn diagram was constructed to illustrate overlapping transcripts among all four comparisons (Fig. 2B). Within each main comparison, the overlap from sub-categories was subtracted from the total number of diet- or genotype-regulated transcripts (totals shown in Table 1). Only six transcripts were found in common among all four comparisons and of these, only three were annotated: *Dnajb9* (DnaJ homolog, subfamily B, member 9), *Oas1c* (2′-5′ oligoadenylate synthase 1C), and *Prlhr* (prolactin releasing hormone receptor).

### Gene expression in HF-fed *Acads−/−* mice resembles that of *Acads+/+* mice on LF diet

**Figure 3:**
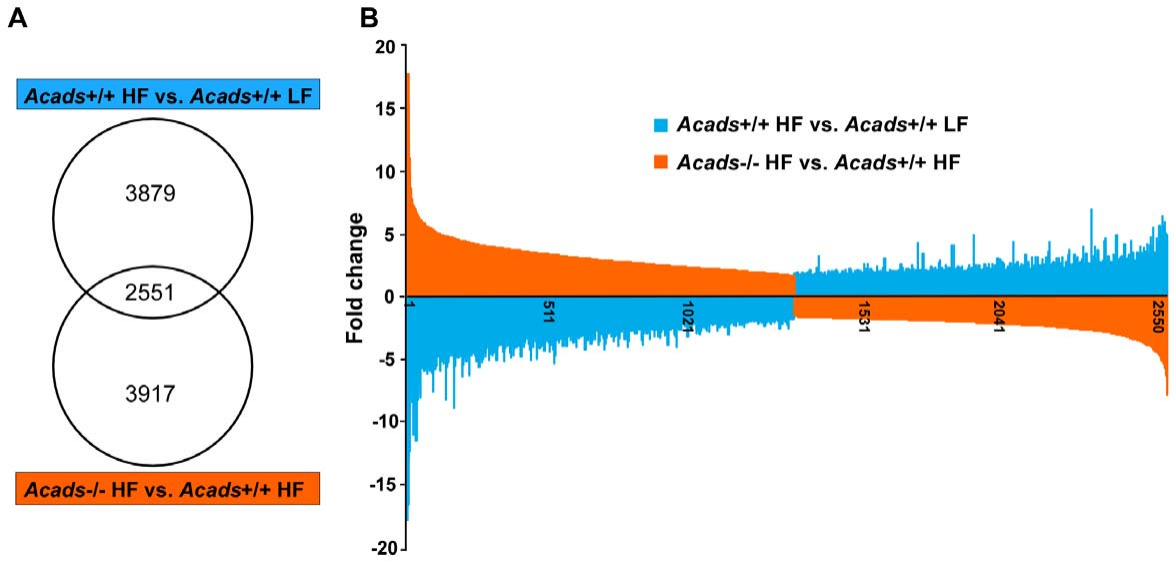
Transcriptional responses of *Acads−/−* mice fed HF diet are similar to those of *Acads+/+* mice fed LF diet. A: Comparison of statistically significant responses from HF *Acads−/−* vs. HF *Acads+/+* (genotype effects) and from HF *Acads+/+* vs. LF *Acads+/+* mice (diet effects) revealed that 2551 genes or approximately 65% were differentially expressed in both experimental comparisons (X-axis). B: All but one of the 2551 genes were expressed in opposite direction, as indicated (blue vs. tan color), and with similar magnitude of change (Y-axis).

When we compared *Acads+/+* to *Acads−/−* mice on HF diet, 3917 genes were differentially expressed based on our study criteria (fold-change above 1.6 and *P*<0.05) (Table 1). A comparison of HF to LF diet in *Acads+/+* controls revealed 3879 differentially expressed genes. An examination of both sets of results revealed that they share 2551 genes or approximately 65% in common (Fig. 3A). On further evaluation, we discovered that 2550 of these genes were expressed in opposite direction in one result set versus the other, and with similar magnitude (Fig. 3B). For example, genes whose expression increased in wildtype controls as an effect of diet (HF vs. LF), were decreased in HF diet as an effect of genotype (*Acads−/−* vs. wildtype). This finding demonstrates that the response to HF diet in *Acads*-deficient mice yields transcriptional responses similar to those of wildtype *Acads+/+* animals on LF diet, i.e., that *Acads−/−* mice show a diminished response to HF diet.

An alternative hypothesis is that *Acads−/−* on LF diet show a HFD-like transcriptome. However, an examination of the result sets for two comparisons: *Acads−/−* HF to LF diet (281 genes) and *Acads+/+* HF to LF (3879) genes revealed only 41 genes in common (results not shown). Of these, only 23 genes showed increased expression in both wildtype and mutant animals as a result of HF diet, lending minimal support to this hypothesis.

### Ingenuity canonical pathway analysis

**Figure 4:**
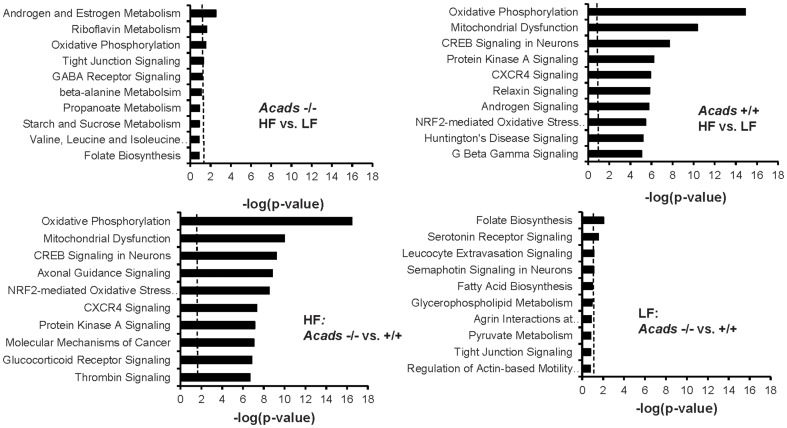
Top ten pathways disturbed in brain by *Acads* genotype and dietary fat. Classification of differentially expressed genes (DEG) according to the top ten pathways most significantly affected by *Acads* genotype or dietary fat level in the brain using Ingenuity Pathways Analysis (IPA). Bars indicate the likelihood [−log (*P*-value)] that the specific category was affected by *Acads* deficiency or dietary fat level compared with others represented in the list of DEG. The vertical dotted line represents the threshold value for significance of *P*<0.05 (1.301 −log scale).

Ingenuity Pathway Analysis (IPA) was used to further organize the differentially expressed genes into functionally annotated pathways and networks. The most significant pathways affected by either genotype or dietary fat are shown in Fig. 4. Two comparisons (*Acads−/−*HF vs *Acads+/+*HF and *Acads+/+*HF vs *Acads+/+*LF) shared the same three top-scoring pathways including oxidative phosphorylation, mitochondrial dysfunction, and CREB signaling in neurons. The oxidative phosphorylation pathway includes mitochondrial complex genes such as NADH dehydrogenases, succinate dehydrogenase, cytochrome c oxidases (COX) and ATP synthases, as well as ubinquinol-cytochrome c reductases and ATPases. The mitochondrial dysfunction pathway contains glutathione, glutaredoxin, and glutathione reductase. The CREB signaling in neurons pathway ranked 3^rd^ highest, encompassing differentially expressed genes such as adenylate cyclases, cAMP responsive element binding protein 5 (CREB5), guanine nucleotide binding proteins, glutamate receptors, MAPK, phosphoinositide kinases, and protein kinase C. In total, 142 pathways were significantly affected by genotype in HF diet, compared with only 2 in LF diet. Three pathways were significantly affected by fat level in *Acads−/−* mice (androgen and estrogen metabolism; riboflavin metabolism; oxidative phosphorylation), in contrast to 114 pathways in *Acads+/+* mice.

### AMP-activated protein kinase (AMPK) related genes

Pathway analysis of our microarray results revealed that AMPK signaling was significantly (5.93×10^−4^) altered in two key experimental comparisons: HF *Acads−/−* vs. HF *Acads+/+* and *Acads+/+* HF vs. LF *Acads+/+*. A set of 18 significantly regulated genes known to act in concert with AMPK are shown in Table 2. For example, gene expression of *Stk32c*, an upstream activator of AMPK as well as *Prkag1*, the protein kinase AMP-activated gamma 1 non-catalytic subunit for AMPK, were increased in HF *Acads−/−* compared to HF *Acads+/+* but were decreased in HF *Acads+/+* vs. LF *Acads+/+*. The activation of AMPK stimulates fatty acid oxidation by inactivating acetyl-CoA carboxylase, and we observed that *Acaca* (acetyl-CoA carboxylase alpha) expression was significantly decreased in HF *Acads−/−* vs. HF *Acads+/+*. These gene expression changes suggest that in *Acads*-deficient animals, a perceived deficit in the amount of energy obtained from dietary fat activates the cellular energy sensor AMP-activated protein kinase pathway [17]. By contrast, each of these genes in wildtype controls (HF vs. LF) is expressed in opposite direction demonstrating once again that HF *Acads−/−* mice respond to HF diet in a manner similar to wildtype controls on LF diet.

**Table 2:** Differentially expressed AMP-kinase related genes in microarray of brain.

			*Acads−/−*HF vs *Acads+/+*HF		*Acads+/+* HF vs *Acads+/+*LF	
Probe ID	Gene Symbol	Gene Name	Fold change	*P* value	Fold change	*P* value
**Stk11 (upstream activator of AMPK):**						
880933	Stk32c	serine/threonine kinase 32C	2.82	0.0292	−2.74	0.0347
437305	Stk38	serine/threonine kinase 38	−5.72	0.0295	5.19	0.0181
561884	Stk11ip	serine/threonine kinase 11 interacting protein	−1.36	0.0446	1.50	0.0250
773189	Stk38l	serine/threonine kinase 38 like	−1.54	0.0140	1.50	0.0168
342708	Stk10	serine/threonine kinase 10	−1.64	0.0182	1.44	0.0329
**AMPK:**						
861185	Prkag1	protein kinase, AMP-activated, gamma 1 non-catalytic subunit	1.25	0.0467	−1.28	0.0175
776294	Ampd3	AMP deaminase 3	−5.51	0.0100	4.45	0.0214
470378	Akap8	A kinase (PRKA) anchor protein 8	−1.96	0.0258	1.95	0.0251
558457	Akap6	A kinase (PRKA) anchor protein 6	−1.81	0.0053	1.81	0.0059
866474	Akap5	A kinase (PRKA) anchor protein 5	−2.52	0.0243	1.64	0.0201
729846	Prkar1b	protein kinase, cAMP dependent regulatory, type I beta	−1.57	0.0304	1.86	0.0186
743151	Prkar2b	protein kinase, cAMP dependent regulatory, type II beta	−1.89	0.0048	1.79	0.0378
423852	Acss2	acyl-CoA synthetase short-chain family member 2	−3.37	0.0172	2.64	0.0069
655175	Creb5	cAMP responsive element binding protein 5	−1.96	0.0017	2.29	0.0093
532068	Arpp21	cyclic AMP-regulated phosphoprotein, 21	−1.96	0.0046	2.33	0.0074
304484	Rapgef4	Rap guanine nucleotide exchange factor (GEF) 4	−1.84	0.0418	2.07	0.0387
814684	Akap6	A kinase (PRKA) anchor protein 6	−4.29	0.0458	1.81	0.0059
**ACC (downstream target of AMPK):**						
438015	Acaca	acetyl-Coenzyme A carboxylase alpha	−1.94	0.0263	1.67	0.0612

Positive fold change indicates increased expression, negative value indicates decreased expression in the first strain of the comparison. Genes were selected based on a significance level of *P*<0.05. Stk11, serine/threonine kinase 11 (synonym Lkb1); AMPK, adenosine monophosphate-activated protein kinase; ACC, acetyl-CoA carboxylase.

### Increased hypothalamic pAMPK in Acads−/− mice fed HF diet

**Figure 5:**
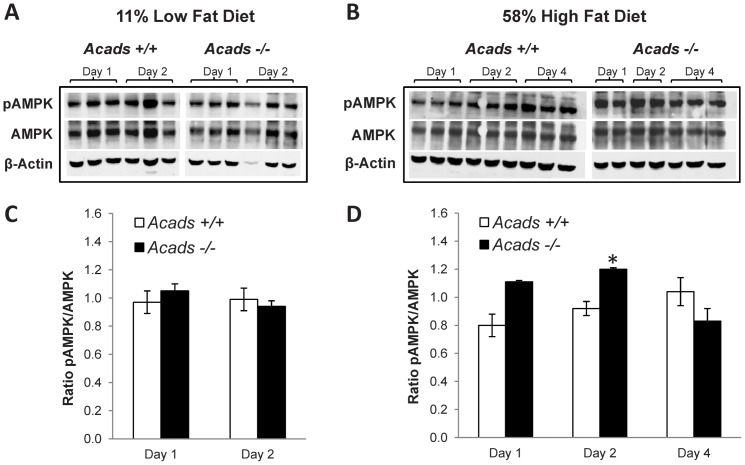
Effects of diet, genotype and day on pAMPK in the hypothalamus. On low-fat diet, there was no effect of genotype on pAMPK level in hypothalamus (A, C). On Day 2 of high-fat diet, *Acads−/−* mice showed increased pAMPK levels compared to *Acads+/+* mice (B, D). Representative immunoblots for total AMPK, pAMPK, and beta actin are shown. *n* = 2–3 per genotype and day. *P<0.05 vs. *Acads+/+*.

The integration of extracellular hormonal and nutrient signals involves hypothalamic AMPK activity, a key signal in the response to changes in energy balance [17]–[18] including consumption of high-fat diet [19]. Immunoblot analysis of hypothalamic tissue showed enhanced AMP kinase activity as evidenced by a 20% increase in (Thr-172) phosphorylation of the AMPK protein in *Acads*-deficient mice fed HF diet for 2 d, compared with controls (Fig. 5).

### Neuropeptide and neurotransmitter genes

Pathway analysis uncovered statistically significant changes in expression of neuropeptide and neurotransmitter genes known to play a role in the regulation of food intake (Table 3). Most of these transcripts were expressed in opposite direction and with similar magnitude, i.e., expression was increased when comparing HF-fed mutant to HF-fed wildtype, and decreased in wildtype controls on LF (relative to wildtype on HF). For example, gene expression of the orexigenic peptides *Npy* (neuropeptide Y) and *Gal* (galanin) was increased in HF-fed *Acads−/−* mice compared with *Acads+/+* (Table 3). The increased expression of *Cart* (cocaine- and amphetamine-regulated transcript) in HF-fed *Acads−/−* mice is surprising, but is consistent with evidence for an orexigenic role of this neuropeptide [20]–[23]. In addition, other genes involved in the control of food intake were differentially expressed such as *Nts* (neurotensin), *Gabbr1* (gamma-aminobutyric acid B receptor, 1), several genes related to GABA-A receptor activity (7 genes), dopamine (6 genes), and opioids (3 genes) (Table 3). Results for *Agrp* and *Ghrelin* were not obtained in this microarray analysis because they did not meet the signal/noise ratio specification.

**Table 3:** Differentially expressed neuropeptide and neurotransmitter genes in microarray of brain.

		*Acads−/−*HF vs *Acads+/+*HF		*Acads+/+* HF vs *Acads+/+*LF	
Gene or related gene name	Gene symbol	Fold change	*P*-value	Fold change	*P*-value
Neuropeptide Y	Npy	3.41	0.0477	−3.77	0.0380
CART	Cart	3.05	0.0311	−4.10	0.0177
Hypocretin (Orexin)	Hcrt	3.33	0.0331	−3.75	0.0434
Galanin	Gal	3.16	0.0141	−2.99	0.0179
Serotonin	Fcgr3	1.84	0.0312	−1.53	0.0469
Neuropeptide W	Npw	2.72	0.0182	−2.64	0.0202
Neurotensin	Nts	2.65	0.0260	−2.57	0.0347
MCH	Ddt	3.78	0.0127	−3.03	0.0364
GABA	Gabbr1	−4.37	0.0041	2.64	0.0012
	Gabra1	−1.96	0.0022	1.84	0.0000
	Gabrb1	−1.28	0.0459	1.38	0.0024
	Gabrb2	−2.15	0.0164	1.71	0.0439
	Gabrg2	−1.62	0.0090	1.83	0.0038
	Gabrb3	−2.00	0.0074	1.98	0.0178
	Gabra3	−2.39	0.0041	1.94	0.0092
	Gabarapl1	−2.54	0.0195	2.35	0.0192
	Slc32a1	−2.02	0.0054	2.14	0.0012
	Slc6a11	−1.49	0.0205	1.59	0.0200
Dopamine	Gnao1	−3.10	0.0135	2.53	0.0293
	Sncaip	−3.08	0.0428	1.63	0.0455
	Nsg2	−1.91	0.0107	1.74	0.0020
	Aldh5a1	−1.81	0.0168	1.63	0.0402
	Adcy5	−1.78	0.0224	2.05	0.0203
	Adora2a	1.37	0.0001	−1.30	0.0224
Opioid	Klf16	1.40	0.0264	−1.45	0.0103
	Opcml	2.70	0.0240	−2.62	0.0148
	Ogfrl1	−1.75	0.0432	2.07	0.0252

Transcriptional responses of *Acads−/−* mice fed HF diet were similar to those of *Acads+/+* mice fed LF diet. Fold change indicates positive (increased) or negative (decreased) expression, relative to the first strain of the comparison. Genes were selected based on a significance level of *P*<0.05 and ≥1.6-fold change in expression, i.e., ratio of the normalized signal intensity.

### Real-time RT-PCR validation of microarray results in hypothalamus

Using quantitative RT-PCR in hypothalamus, we examined a subset of fatty acid metabolism genes that were differentially expressed in the microarrays, as well as some neuropeptide genes (Table 4). Several genes involved in fatty acid beta-oxidation were coordinately down-regulated in HF-fed *Acads*-deficient mice compared to wildtype. Significantly decreased expression was found for *Cox8b* (cytochrome c oxidase, subunit VIIIb), an enzyme of the mitochondrial respiratory chain; UCP3 a mitochondrial uncoupling protein; *Acox1* and *Cpt1c* encoding beta-oxidation enzymes; *Acaca* (acetyl-CoA carboxylase alpha), and *Fasn* (fatty acid synthase), and *Mlycd* (malonyl-CoA decarboxylase) which are involved in fatty acid biosynthesis. *Creb5* is a cAMP-responsive transcription factor with a newly described role in the regulation of hypothalamic metabolism [24] and energy regulation [25]. Approximately one-half of the qPCR results confirmed those of the microarray analysis (Table 4). Although changes in RNA expression do not necessarily correspond to changes in protein expression, the overall pattern of these related genes suggests a functional change.

**Table 4:** Quantitative RT-PCR for neuropeptide and energy sensing genes in hypothalamus.

Experimental comparison:		*Acads−/−*HF vs *Acads+/+*HF		*Acads+/+* HF vs *Acads+/+*LF	
		Fold change	*P*-value	Fold change	*P*-value
Neuropeptides	Agrp	1.06	0.846	−1.42	0.049
	Cart	−1.34	0.227	1.05	0.834
	Npy	−1.09	0.581	−1.22	0.161
	Pomc	−1.46	0.011	1.08	0.722
Energy status	Acaca	−1.31	0.004	−1.17	0.331
	Acox1	−1.21	0.033	−1.07	0.353
	Cox8b	−1.58	0.015	−1.12	0.530
	Cpt1c	−1.06	0.178	−1.01	0.781
	Creb5	−1.07	0.455	−1.02	0.887
	Fasn	−1.30	0.014	1.10	0.249
	Mlycd	−1.27	0.014	1.04	0.748
	Prkag1	−1.05	0.108	1.10	0.144
	Ucp3	−1.12	0.089	−1.05	0.476

Quantitative RT-PCR for selected genes was performed on twelve hypothalamus samples per condition, reactions were done in triplicates. Delta Ct values were determined relative to the Ct value for *Ppia* in the same sample. *P*-values were obtained by performing two-tailed Student's *t*-test on delta Ct values.

The downstream effects of phosphorylated AMPK may be expressed through changes in neuropeptide signaling, therefore we examined orexigenic agouti-related peptide (*Agrp*) and neuropeptide Y (*Npy*) and anorexigenic pro-opiomelanocortin (*Pomc*) and cocaine- and amphetamine-regulated transcript (*Cart*). Using real-time RT-PCR, we observed significant reductions in the anorexigenic peptide *Pomc* in HF fed *Acads−/−* compared with HF *Acads+/+* mice (*P*<0.05) (Table 4), suggesting a compensatory response to negative energy balance in *Acads*-deficient mice. This result contrasts with the significantly decreased expression of orexigenic *Agrp* in wildtype animals (*P*<0.05) (Table 4), which may represent a compensatory response to the positive energy balance associated with acute exposure to HF diet [26].

### PANTHER analysis identified diet-regulated chemosensory processes

We also used PANTHER Analysis to identify processes that were overrepresented in GO Biological Process categories, but not portrayed by pathway analysis tools (Table 5). The reported *P* value was based on the number of differentially expressed genes obtained versus the number that would be expected. Calculation of the number of genes expected was based on 281 genes in *Acads−/−* and 3917 genes in *Acads+/+* mice. Of note, diet-regulated biological processes such as chemosensory perception and olfaction were under-represented in *Acads+/+* mice and by contrast, over-represented in the *Acads−/*− mice, including G-protein signaling, the the primary sensing mechanism for vision, taste and smell. Sensory organs provide information that allows animals to effectively identify foods that have been associated with both positive and negative internal consequences. One possible interpretation of these results is that chemosensory pathways were invoked in *Acads−/−* mice to support associative learning about the negative internal consequences of HF diet, i.e., incomplete oxidization and reduced energy availability.

**Table 5:** Results of PANTHER analysis for olfactory and sensory genes.

*Acads−/−* HF vs LF					
	Mouse	Genes	Genes		
Biological Process	AB genes	Obtained	Expected	Represented	*P*-value
G-protein mediated signaling	1251	27	10.36	+	1.07E-03
Chemosensory perception	463	15	3.83	+	1.28E-03
Olfaction	457	15	3.78	+	1.51E-03
Sensory perception	873	17	7.23	+	3.21E-02

*Acads+/+* HF vs LF					
	Mouse	Genes	Genes		
Biological Process	AB genes	Obtained	Expected	Represented	*P*-value
G-protein mediated signaling	1251	115	183.20	-	3.94E-06
Chemosensory perception	463	24	67.80	-	8.46E-08
Olfaction	457	22	66.92	-	2.38E-08
Sensory perception	873	57	127.84	-	2.24E-11

PANTHER Analysis was used to identify processes that were overrepresented in Gene Ontology Biological Process categories. The reported *P* value was based on the number of differentially expressed genes obtained versus the number that would be expected. Calculation of the number of genes expected was based on 281 genes in *Acads−/−* and 4014 genes in *Acads+/+* mice.

Our data have identified the brain molecular pathways and genes that respond to fat intake in mice with a genetic inactivation of *Acads*, encoding short-chain acyl-CoA dehydrogenase (SCAD). The lack of functional SCAD enzyme in *Acads−/−* mice results in the impaired oxidation of short-chain fatty acids, as demonstrated by the significantly higher levels of C4 and C5 acylcarnitines in plasma of SCAD deficient mice (Fig. 1). Using a whole genome array, changes in gene expression were evaluated in mutant or wildtype mice at 2 d after initiating experimental HF or LF diets. The top three pathways affected by diet or genotype were oxidative phosphorylation, mitochondrial dysfunction, and CREB signaling in neurons. For the majority of genes that map to these pathways, HF-fed *Acads*−/− mice diet displayed transcriptional responses similar to those of wildtype *Acads+/+* mice fed LF diet (Fig. 3). Specifically, genes involved in energy sensing, mitochondrial function and the peptidergic control of feeding behavior followed this pattern. In particular, an examination of the gene expression profile for energy sensing and AMPK-related genes suggests that the decreased beta-oxidation of short-chain fatty acids in *Acads*-deficient mice fed HF diet produces a condition of energy deficiency in the brain (Table 2). Further investigation in hypothalamus using quantitative RT-PCR confirmed the dysregulation of genes in these pathways. Western blotting showed increased hypothalamic AMP-kinase, a key protein in an energy-sensing cascade that responds to depletion of ATP.

Previously we demonstrated that in a diet choice paradigm *Acads−/−* mice, compared to *Acads+/+*, shifted consumption away from fat- and toward carbohydrate-containing diets, thus effectively preventing a reduction in total calories [8]. In particular, *Acads−/−* mice significantly reduced their self-selected fat intake on day two of diet selection providing the rationale for examining gene expression changes on day two after diet initiation in the present study. We hypothesized that gene expression changes at this time-point would reveal functionally important pathways mediating the behavioral feeding response to dietary fat.

The LF and HF experimental diets used in the current study are composed mainly of C12–C18 fatty acids (86–91%) and thus provide plenty of long-chain fatty acids for beta-oxidation. SCAD deficiency blocks only the beta oxidation of C4–C6 substrates, resulting in nearly normal amounts of acetyl-CoAs along with the accumulation of butyric acid. We speculate that the incomplete oxidation of short-chain fatty acids due to SCAD deficiency may have produced a small mitochondrial oxidative deficiency, consistent with a previous report of 20% lower acetyl-CoA levels in the brain of *Acads−/−* compared with *Acads+/+* mice [27].

Results from the microarray revealed the significant down-regulation of genes involved in the activation of AMP-kinase in *Acads−/−* mice compared with *Acads+/+* on HF diet, suggesting that impaired SCFA oxidation resulted in energy deficiency. Because hypothalamic AMP-kinase is decreased in mice fed high-fat diet [19], we also examined the effects of *Acads* deficiency on hypothalamic AMPK in *Acads−/−* mice. We hypothesized that the reduced cellular energy leading to expression changes in AMPK-related and neuropeptide genes could ultimately be responsible for the altered macronutrient diet selection observed previously in *Acads−/−* mice [8].

The brain possesses a high density of mitochondria which are needed to provide energy to maintain cell functions [28]. Although the brain relies primarily on glucose metabolism for energy, it can also use ketones formed in the liver under conditions of low carbohydrate availability. Normally, cytosolic fatty acyl-CoAs taken up by mitochondria undergo beta-oxidation or are converted into various structural and signaling lipid molecules [29]. Inside the mitochondria, fatty acids undergo beta-oxidation in a process whereby acyl-CoA dehydrogenases, differing in their substrate specificity based on the fatty acid chain length, cleave two carbons at a time from acyl-CoA molecules starting at the carboxyl end, e.g., ACADS catalyzes the first step in the beta-oxidation of C_4_–C_6_ fatty acids. The two-carbon units of acetyl-CoA then enter the Krebs cycle and provide substrates for gluconeogenesis and ketogenesis. Although fatty acid oxidation in the brain is modest, it is important in determining the metabolic fate of fatty acids that have entered cerebral tissue [29]–[30].

Whether or not the observed transcriptional effects in the brain of *Acads−/−* mice were the result of *Acads* deficiency within or outside of the brain remains in question. The evidence for a primary role of fatty acid oxidation in ATP production in brain cells is quite scant. Nevertheless it remains possible that a very small amount of fatty acid beta-oxidation in the brain may function to generate energy [29], [31]–[33] in addition to other functions such as the synthesis and/or degradation of unique cellular lipids for neuronal function [34]. The fact that both ACADS mRNA and protein are expressed in several distinct brain nuclei [9] and that MCAD is highly expressed in neuronal axons and dendrites [34] suggests a primary role for beta-oxidation in ATP production in these cells. Moreover, fatty acid oxidation enzyme activity, although low, is measurable in whole brain; for example in SCHAD (short chain 3-hydroxyacyl-CoA dehydrogenase) knockout mice, enyzme activity was lost in an allele-specific manner with heterozygotes showing 50% of the activity of wildtype animals (Dr. Michael J. Bennett, personal communication). However, it is not yet known which cell types in the brain express these enzymes. Therefore clear evidence that brain cells use fatty acids as an oxidative source of energy production awaits future studies.

**Figure 6:**
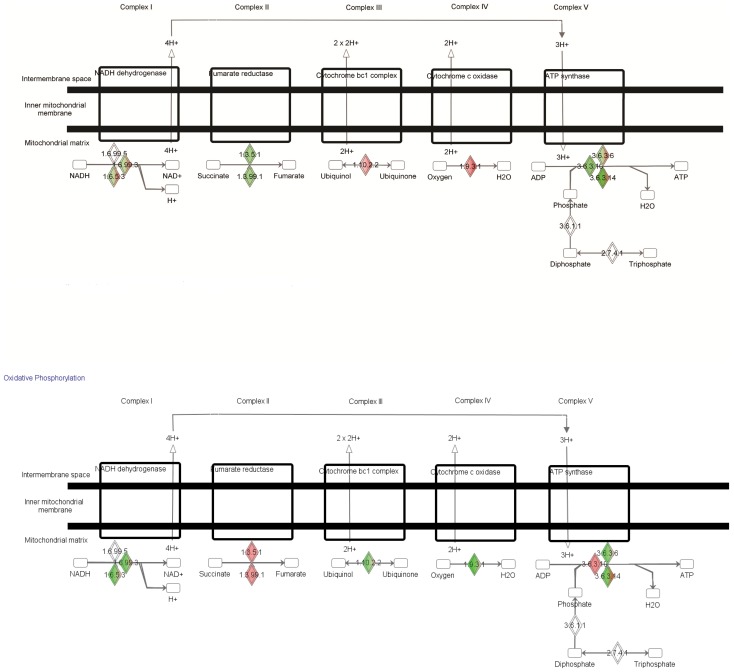
IPA analysis reveals oxidative phosphorylation pathway. Shown are the results from the IPA functional annotation after analysis of all the genes with a fold change value of equal or greater than 1.6 and *P* value of <0.05. Up-regulated genes are colored in red, down-regulated genes in green. The diamond shapes with both colors contain gene families in which some genes are up-regulated, and some are down-regulated. Overall, the oxidative phosphorylation pathway was the most significantly activated pathway in the brain of HF *Acads−/−* (ratio 0.414) vs. HF *Acads+/+*, and of HF *Acads+/+* vs. LF *Acads+/+* (ratio 0.393) mice. Notably in HF *Acads−/−* vs. HF *Acads+/+* mice, we found significantly decreased expression of ATP synthase genes in mitochondrial complex 5 (top panel), consistent with a state of decreased energy availability.

Pathway analysis identified oxidative phosphorylation as one of the top three pathways that were significantly activated by genotype in HF-fed mice (Fig. 4). The oxidative phosphorylation and electron transport carried out by the respiratory chain in the mitochondria is composed of five complexes that lead to the production of ATP. In our microarray study, expression of genes representing all five mitochondrial complexes was significantly altered by SCAD deficiency and HF diet. For example in HF *Acads−/−* vs. HF *Acads+/+* mice, we found significantly decreased expression of ATP synthase genes in mitochondrial complex 5 (Fig. 6A), thus providing evidence for decreased energy availability.

Mitochondrial oxidative phosphorylation and resulting ATP levels in the brain play a key role in the control of feeding. The observation of significant changes in gene expression of neuropeptides and transmitters in whole brain led us to examine hypothalamic expression of selected genes involved in the metabolic control of food intake. For example, proopiomelanocortin is the precursor of melanocortin peptides synthesized in the arcuate nucleus of the hypothalamus and is an important regulator of food intake. The reduced hypothalamic *Pomc* expression in *Acads*-deficient animals fed HF diet is reminiscent of the response to fasting [35] providing further evidence of decreased energy availability.

The gene expression results of the current study revealed an over-representation of the biological processes chemosensory perception and olfaction in *Acads−/−* mice as an effect of diet (Table 5). It is tempting to speculate that these pathways are involved in associative learning, e.g., that gene expression in the CNS was altered when the post-oral consequences of the diet were associated with their sensory characteristics in a memory forming process [36]. Learning about the energy consequences of food enables animals to detect and distinguish between food sources in subsequent encounters, both in the wild and in laboratory settings. We propose that the behavior of *Acads−/−* mice to avoid a fat-based diet and increase consumption of a carbohydrate-rich diet [8] may involve such learning.

## Conclusions

**Figure 7:**
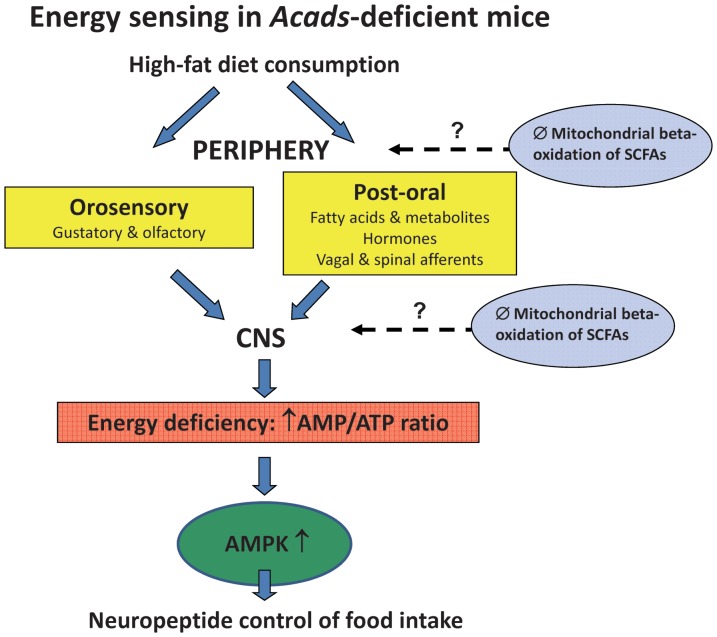
Energy-sensing in *Acads*-deficient mice. Schematic flow diagram showing possible neural processing of cues from incomplete oxidation of high-fat diet (HFD) leading to changes in food intake. In *Acads*-deficient mice, a lack of mitochondrial SCFA beta-oxidation has been demonstrated in both peripheral and central tissues [9] which could generate signals that contribute to energy-sensing mechanisms. Orosensory and post-oral signals generated in the periphery in response to HFD are integrated within the CNS, e.g., neurons within the hypothalamus respond to changes in energy availability. Impaired oxidation of SCFAs within the brain could also signal an energy deficiency. Hypothalamic AMPK is then activated by an increase in the AMP-to-ATP ratio, which reflects the energy status of the cell [37] and induces reciprocal modifications of neuropeptides expression and food intake [18]. Abbreviations: SCFA, short chain fatty acid; CNS, central nervous system; ATP, adenosine-5′-triphosphate; AMPK, adenosine monophosphate-activated protein kinase.

We found altered brain expression of genes involved in energy production and energy sensing. Remarkably, the subset of genes deregulated in HF-fed *Acads−/−* mice showed an expression pattern that strongly resembled that of *Acads+/+* mice on LF diet, in particular, genes involved in both oxidative phosphorylation and AMP-kinase signaling. The differential expression of brain peptide and transmitter genes known to control food intake suggest possible downstream targets leading to changes in feeding behavior (Fig. 7). Therefore these genes could be responsible for the altered feeding behaviors observed in *Acads−/−* mice.

## Supporting Information

Figure S1 **Functional categories of genes with altered expression as a function of genotype or dietary fat.** Transcripts were sorted based on gene ontologies assigned in the MGI database version 3.0. The majority of diet-regulated genes in *Acads*-deficient mice (54.2%) fell into four categories: unknown (21.4%), signal transduction (13.9%), protein metabolism (10.0%), and developmental processes (8.9%), while those altered in *Acads*-replete mice (42.2%) were: transport (11.8%), developmental processes (10.3%), signal transduction (10.1%), and protein metabolism (10.0%). The majority of genes regulated by genotype on HF diet fell into categories of: transport (12%), developmental processes (10.6%), signal transduction (10.3%), and protein metabolism (10.1%). Those categories most affected by genotype with LF diet consisted of: unknown (36.4%), signal transduction (11.3), transport (8.0%), and developmental processes (5.8%).
(TIFF)

Table S1 **Macronutrient and fatty acid composition of the experimental diets.**
(DOC)

Table S2 **Primer sequences for quantitative RT-PCR.**
(DOC)

Table S3 **Analysis of variance results for acylcarnitines.**
(DOC)

Table S4 **Quantitative real-time RT-PCR validation in whole brain of microarray data for selected genes in the experimental comparison:** ***Acads−/−*** **HF vs.** ***Acads+/+*** **HF.**
(DOC)

Text S1 **Quantitative real-time RT-PCR validation of microarray data for selected genes.**
(DOC)
