# Supplementary figures and images for: Brain Transcriptional Responses to High-Fat Diet in Acads-Deficient Mice Reveal Energy Sensing Pathways
Source: PLoS One. 2012 Aug 22;7(8):e41709. doi: 10.1371/journal.pone.0041709 (PMC3425564; doi:10.1371/journal.pone.0041709)

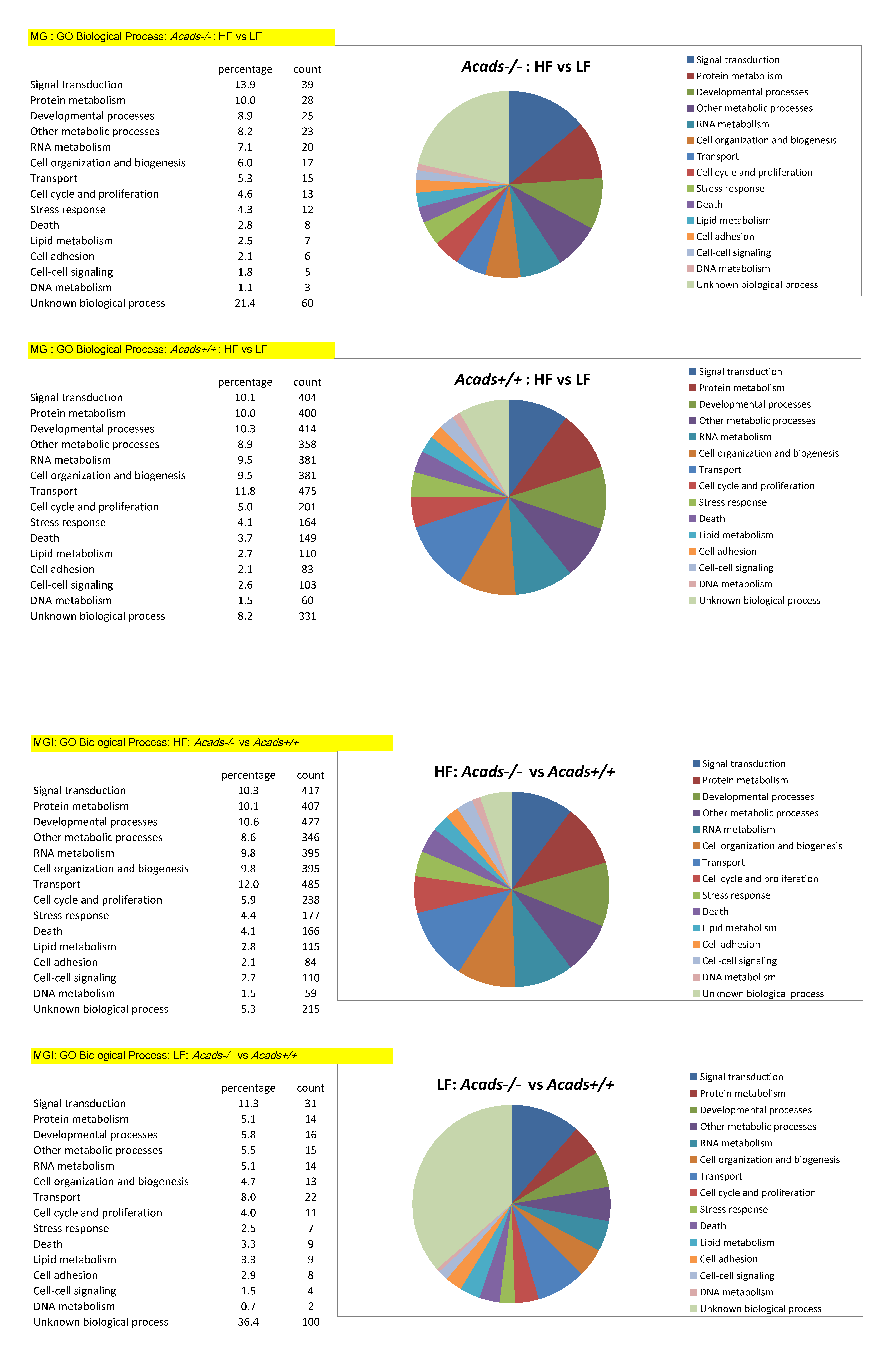

Supplement: Figure S1 — Functional categories of genes with altered expression as a function of genotype or dietary fat. Transcripts were sorted based on gene ontologies assigned in the MGI database version 3.0. The majority of diet-regulated genes in Acads-deficient mice (54.2%) fell into four categories: unknown (21.4%), signal transduction (13.9%), protein metabolism (10.0%), and developmental processes (8.9%), while those altered in Acads-replete mice (42.2%) were: transport (11.8%), developmental processes (10.3%), signal transduction (10.1%), and protein metabolism (10.0%). The majority of genes regulated by genotype on HF diet fell into categories of: transport (12%), developmental processes (10.6%), signal transduction (10.3%), and protein metabolism (10.1%). Those categories most affected by genotype with LF diet consisted of: unknown (36.4%), signal transduction (11.3), transport (8.0%), and developmental processes (5.8%). (TIFF) [file pone.0041709.s001.tiff]
