# Supplementary material for: Brain Transcriptional Responses to High-Fat Diet in Acads-Deficient Mice Reveal Energy Sensing Pathways
Source: PLoS One. 2012 Aug 22;7(8):e41709. doi: 10.1371/journal.pone.0041709 (PMC3425564; doi:10.1371/journal.pone.0041709)
Supplement: Table S2 — Primer sequences for quantitative RT-PCR. (DOC) [file pone.0041709.s003.doc]

Table S1: Primers used for quantitative real-time PCR assays.

| Gene symbol | Accession # | Forward primer - sequence | Position | Reverse primer -sequence | Position | AE |
| --- | --- | --- | --- | --- | --- | --- |
| Agrp | [NM_007427](http://www.ncbi.nlm.nih.gov/nuccore/NM_007427.2) | 5'-AGCTTTGGCGGAGGTGCTA-3' | 201 | 5'-AGGACTCGTGCAGCCTTACAC-3' | 277 | 1.88 |
| Cart | [NM_013732](http://www.ncbi.nlm.nih.gov/nuccore/NM_013732.6) | 5'-ACGAGAAGAAGTACGGCCAAGT-3' | 254 | 5'-CCGATCCTGGCCCCTTT-3' | 329 | 1.78 |
| Npy | [NM_023456](http://www.ncbi.nlm.nih.gov/nuccore/NM_023456.2) | 5'-CCGCTCTGCGACACTACATC-3' | 149 | 5'-AATCAGTGTCTCAGGGCTGGAT-3' | 222 | 1.83 |
| Pomc | [NM_008895](http://www.ncbi.nlm.nih.gov/nuccore/NM_008895.3) | 5'-CTCCTGCTTCAGACCTCCATAGA-3' | 46 | 5'-GGATGCAAGCCAGCAGGTT-3' | 142 | 1.92 |
| Acaca | [NM_133360](http://www.ncbi.nlm.nih.gov/nuccore/NM_133360.2) | 5'-ATGTCCGCACTGACTGTAACCA-3' | 4391 | 5'-TGCTCCGCACAGATTCTTCA-3' | 4480 | 1.82 |
| Acox1 | [NM_015729](http://www.ncbi.nlm.nih.gov/nuccore/NM_015729.2) | 5'-AGCCTCTGCCAGGCATCAC-3' | 647 | 5'-CATCAACATGTTCTCTCTAGGAATACG-3' | 762 | 1.98 |
| Cox8b | [NM_007751](http://www.ncbi.nlm.nih.gov/nuccore/NM_007751.3) | 5'-CCATAGTCGTTGGCTTCATGGT-3' | 134 | 5'-GCTGCGGAGCTCTTTTTATAGC-3' | 209 | 1.99 |
| Cpt1c | [NM_153679](http://www.ncbi.nlm.nih.gov/nuccore/NM_153679.1) | 5'-GACAACAAGGAGACAGACCAACAT-3' | 1846 | 5'-CATCGCCGCCTTCAGTAGAG-3' | 1929 | 1.84 |
| Creb5 | [NM_172728](http://www.ncbi.nlm.nih.gov/nuccore/NM_172728.2) | 5'-GACTTCGTAAGCTCCAACTGTGAA-3' | 2012 | 5'-GGCCATAGAAGCGAGCTGTAA-3' | 2097 | 1.87 |
| Fasn | [NM_007988](http://www.ncbi.nlm.nih.gov/nuccore/NM_007988.3) | 5'-GGCACTGACTGTCTGTTTTCCA-3' | 8129 | 5'-TGTAAAAATGACACAGTCCAGACACTT-3' | 8228 | 1.86 |
| Mlycd | [NM_019966](http://www.ncbi.nlm.nih.gov/nuccore/NM_019966.2) | 5'-CGCTGCCATCTTCTACTCCAT-3' | 849 | 5'-AATTCCTTCTGCAGCTCCTTGA-3' | 953 | 1.92 |
| Ucp3 | [NM_009464](http://www.ncbi.nlm.nih.gov/nuccore/NM_009464.3) | 5'-CCACCTTAGGGCAAGAACGA-3' | 1836 | 5'-AGATGAGAAAACCTCCGAGAGAGA-3' | 1899 | 1.96 |
| 1700030F18Rik | [NM_028180](http://www.ncbi.nlm.nih.gov/nuccore/NM_028180.3) | 5'-CCTTCAGAAAATCTCTTGCTTCCA-3' | 260 | 5'-TGGGTTTTGTGGTCACTTACGA-3' | 327 | 1.95 |
| Acad10 | [NM_028037](http://www.ncbi.nlm.nih.gov/nuccore/NM_028037.4) | 5'-GCCTGGCCGGATTCATC-3' | 2745 | 5'-GGACATCACCCGTGTCTTCAT-3' | 2826 | 1.90 |
| AW112010 | [NM_001177351](http://www.ncbi.nlm.nih.gov/nuccore/NM_001177351.1) | 5'-TGCAACAATACCTGGCGTATAAGT-3' | 56 | 5'-CTGGTATATATGTACATCATTGGCTTGA-3' | 138 | 1.86 |
| Clic6 | [NM_172469](http://www.ncbi.nlm.nih.gov/nuccore/NM_172469.3) | 5'-GGCTAAAGGGTGTTATATTCAATGTG-3' | 1169 | 5'-CAGGAGCCAGGTTCTGAAGGT-3' | 1246 | 1.84 |
| Dock5 | [NM_177780](http://www.ncbi.nlm.nih.gov/nuccore/NM_177780.3) | 5'-CCCTGGAGAGTACAGTGCAATATG-3' | 6365 | 5'-CACCCTAATTTTTCCTTCTGAAAGG-3' | 6478 | 1.90 |
| F2rl2 | [NM_010170](http://www.ncbi.nlm.nih.gov/nuccore/NM_010170.4) | 5'-GCTGCCTAGATCCATTCCTTTACT-3' | 1049 | 5'-CATTGCTGACTAAGGATTAAGCTGAT-3' | 1119 | 1.93 |
| Gpr61 | [NM_175470](http://www.ncbi.nlm.nih.gov/nuccore/NM_175470.4) | 5'-TGGTAACCTGGATTGGCTACTTTT-3' | 1574 | 5'-TGACGGTTGAGACATCCGTAGA-3' | 1640 | 1.70 |
| Neurog3 | [NM_009719](http://www.ncbi.nlm.nih.gov/nuccore/NM_009719.6) | 5'-ACGGCCTCATTGGAGGAATT-3' | 553 | 5'-CCAGACAGGTCTCTTCACAAGAAG-3' | 659 | 1.84 |
| Plekha2 | [NM_031257](http://www.ncbi.nlm.nih.gov/nuccore/NM_031257.3) | 5'-GGTCAAGTCTGGTGATCTCTTAATGA-3' | 762 | 5'-GCTGTCCGCCTGTACGTAGAA-3' | 846 | 1.86 |
| Prlhr | [NM_201615](http://www.ncbi.nlm.nih.gov/nuccore/NM_201615.2) | 5'-CCGCCGCACTTTCTGTCT-3' | 819 | 5'-GTCTCGCAATAGGTTGAAAATGTG-3' | 906 | 1.91 |
| Ptgfr | [NM_008966](http://www.ncbi.nlm.nih.gov/nuccore/NM_008966.3) | 5'-TGACAGCCTATCATGAGCCCTAT-3' | 777 | 5'- AAAGCAACGTTTGCCATGTTC-3' | 872 | 1.89 |
| Rassf10 | [NM_175279](http://www.ncbi.nlm.nih.gov/nuccore/NM_175279.3) | 5'-GGGAAGGACTGATGACAGCTAGA-3' | 1451 | 5'-CCATCCAAGTGTTTTCTTTTAGCA-3' | 1521 | 1.95 |
| Slc22a13 | [NM_133980](http://www.ncbi.nlm.nih.gov/nuccore/NM_133980.3) | 5'-TGCAGGGTCCCACAAAACA-3' | 1557 | 5'-CCACCCTCACACTGGAAGTTC-3' | 1630 | 1.99 |
| Prkag1 | [NM_016781](http://www.ncbi.nlm.nih.gov/UniGene/seq.cgi?ORG=Mm&SID=8539433) | 5'-GTGGCTCTGGGCATCTTTGT-3' | 640 | 5'-CGCCCTTTCTCATCCACTACA-3' | 704 | 1.95 |
| 5830454E08Rik | NC_000075 | 5’-TGTCCGTTGCTAGTAGGATATGCT-3' | 463 | 5’-GCTGGCCTCAACTCCTGATATT-3' | 525 | 1.81 |
| Acot11 | [NM_025590](http://www.ncbi.nlm.nih.gov/UniGene/seq.cgi?ORG=Mm&SID=9104410) | 5’-CCTGGGACCCTAGCAACCA-3' | 1196 | 5’-GGCGGACCTCGCTGATCT-3' | 1306 | 2.01 |
| Aplp2 | [NM_001102455](http://www.ncbi.nlm.nih.gov/UniGene/seq.cgi?ORG=Mm&SID=40598069) | 5’-TGTACAAAGTTCCTTATGTTGCTCAA-3' | 1640 | 5’-TCCATATCCGCTCGCTGTTC-3' | 1718 | 1.76 |
| Arhgap20 | [NM_175535](http://www.ncbi.nlm.nih.gov/UniGene/seq.cgi?ORG=Mm&SID=15343582) | 5’-CACTCATTGGACACGAGTATCCTTA-3' | 767 | 5’-GCTCCATGAGGAAAGGCTCTT-3' | 892 | 1.83 |
| Atp7a | NM_001109757 | 5’-GCTCATAAGGTAAAGGTAGTGGTATTTGA-3' | 3079 | 5’-GGATCTTATTGCGTGATATCTTGTTACT-3' | 3196 | 1.76 |
| Cask | [NM_009806](http://www.ncbi.nlm.nih.gov/UniGene/seq.cgi?ORG=Mm&SID=8041314) | 5’-GGATGAGCCAATGGGAATCA-3' | 1473 | 5’-TCGGATTTCATCACCAACATGA-3' | 1593 | 1.70 |
| Col4a3bp | NM_023420 | 5’-GATAGTTTGTAATTTTTCTGTGGATCATG-3' | 1578 | 5’-TGATTTTGGCACGGACACAT-3' | 1648 | 1.88 |
| Fggy | NM_001113412 | 5’-CGCTGTCTCAGGACCTTGATG-3' | 1238 | 5’-GAAGCGAGTCCCAAAAGCAAT-3' | 1314 | 1.86 |
| Gm941 | [NM_001145452](http://www.ncbi.nlm.nih.gov/UniGene/seq.cgi?ORG=Mm&SID=50822318) | 5’-CGCCTACCTGAGGGATTTACC-3' | 843 | 5’-ATCACCCTCCTTCAGTACCACAA-3' | 909 | 1.80 |
| Gpr137c | [NM_027518](http://www.ncbi.nlm.nih.gov/UniGene/seq.cgi?ORG=Mm&SID=15645396) | 5’-TTTCCGGGCACAGAGATTAAA-3' | 993 | 5’-AGCTGTAGCTGTGGCTGCTTATC-3' | 1060 | 1.84 |
| Gpr44 | [NM_009962](http://www.ncbi.nlm.nih.gov/UniGene/seq.cgi?ORG=Mm&SID=8041430) | 5’-TGGCCTCCAGGTTTCTACACA-3' | 1460 | 5’-TTTACCCGACCCTTATCAGTTACC-3' | 1545 | 1.85 |
| Insrr | [NM_011832](http://www.ncbi.nlm.nih.gov/UniGene/seq.cgi?ORG=Mm&SID=8041801) | 5’-GCTGCCCTCGGTTTCTTCTA-3' | 2812 | 5’-CATACATGTGGGATGCACTGAAAT-3' | 2902 | 1.96 |
| Map3k2 | [NM_011946](http://www.ncbi.nlm.nih.gov/UniGene/seq.cgi?ORG=Mm&SID=8041926) | 5’-CCAGAGACCAGCAAGGAAGTAAA-3' | 1180 | 5’-CGCTCATGCAACAAGTTTTTCA-3' | 1250 | 1.90 |
| Mapk15 | [NM_177922](http://www.ncbi.nlm.nih.gov/UniGene/seq.cgi?ORG=Mm&SID=14960749) | 5’-TGGGCTGCATATTAGGAGAGATG-3' | 614 | 5’-AGCACTGTAGTCTGAGCCAAGGT-3' | 756 | 1.76 |
| Mx2 | [NM_013606](http://www.ncbi.nlm.nih.gov/UniGene/seq.cgi?ORG=Mm&SID=8082272) | 5’-CGCCAGGAGGCTCACAAC-3' | 1732 | 5’-TCAGCAAACATTTTCAGGATGAA-3' | 1808 | 1.87 |
| Ndufc1 | [NM_025523](http://www.ncbi.nlm.nih.gov/UniGene/seq.cgi?ORG=Mm&SID=9104363) | 5’-GGCGCCTCGGTTTTCAT-3' | 145 | 5’-CCAATCCATTTCTTCTTTTATACTCCAA-3' | 226 | 1.89 |
| Npc1 | [NM_008720](http://www.ncbi.nlm.nih.gov/UniGene/seq.cgi?ORG=Mm&SID=8040052) | 5’-CCCTTCGGGCCTCCATT-3' | 1288 | 5’-AGATGCGGTGATGCTTTCAAT-3' | 1368 | 1.80 |
| Nr3c1 | [NM_008173](http://www.ncbi.nlm.nih.gov/UniGene/seq.cgi?ORG=Mm&SID=8040534) | 5’-CATTTGCCCTGGGTTGGA-3' | 1859 | 5’-TCAGATCAGGAGCAAAGCATAGC-3' | 1927 | 1.86 |
| Nrf1 | NM_001164226 | 5’-CGTACCATCACAGACCGTAGTACAG-3' | 900 | 5’-GTGGTTGGCAGTTCTGAAGCA-3' | 1019 | 1.73 |
| Ptprcab | [NM_016933](http://www.ncbi.nlm.nih.gov/nuccore/NM_016933.3) | 5’-CCACCCTTACATCAGCTTCTTAGACT-3' | 683 | 5’-AGTGCCTGTCTGGGATGCA-3' | 759 | 1.95 |
| Qscn6 | NM_001024945 | 5’-AGTTTGCAGACCGCTCCAA-3' | 884 | 5’-TCCCCACTTCTACACGCAAGA-3' | 961 | 1.81 |
| Rpl11 | [NM_025919](http://www.ncbi.nlm.nih.gov/UniGene/seq.cgi?ORG=Mm&SID=9104594) | 5’-CCAAACACAGAATCAGCAAGGA-3' | 458 | 5’-GATCAAGTTTATTTTCCAGGAAGGAT-3' | 545 | 1.87 |
| Sacs | [NM_172809](http://www.ncbi.nlm.nih.gov/UniGene/seq.cgi?ORG=Mm&SID=10828075) | 5’-GCAGACCCGAGGTTATGTGAA-3' | 2181 | 5’-ATTTCCGCTGTATGCCAAAGA-3' | 2253 | 1.87 |
| Sass6 | [NM_028349](http://www.ncbi.nlm.nih.gov/UniGene/seq.cgi?ORG=Mm&SID=23104547) | 5’-GCCACCCCACATTCTACTAGCA-3' | 1480 | 5’-ATGTCAATGCAGAGGAGACAGGAT-3' | 1603 | 1.97 |
| Scn2b | [NM_001014761](http://www.ncbi.nlm.nih.gov/UniGene/seq.cgi?ORG=Mm&SID=25652009) | 5’-GGCCACGGCAAGATTTACCT-3' | 412 | 5’-CTCACACATTTGACCACCATCAG-3' | 551 | 1.88 |
| Tnfsf18 | [NM_183391](http://www.ncbi.nlm.nih.gov/UniGene/seq.cgi?ORG=Mm&SID=16837789) | 5’-CCATCGAGTCCTGCATGGTT-3' | 143 | 5’-GTGAGGTTTGGGAGATGTCATGT-3' | 213 | 2.00 |
| Ugt2b36 | [NM_001029867](http://www.ncbi.nlm.nih.gov/UniGene/seq.cgi?ORG=Mm&SID=26205676) | 5’-ATCTACAAGTGGCTTCCCCAAA-3' | 1060 | 5’-GGATCGCCTCGTAGAGTCCAT-3' | 1156 | 1.84 |
| Cox8a | [NM_007750](http://www.ncbi.nlm.nih.gov/nuccore/NM_007750.2) | 5’-CATCCTTTGACTAGACCACTTTTGC-3' | 228 | 5’-AGGCCAGCCAGGAATGC-3' | 293 | 1.89 |
| Olfml2a | [NM_172854](http://www.ncbi.nlm.nih.gov/nuccore/NM_172854.2) | 5’-TGAGCCACCTTTGCATCCA-3' | 1221 | 5’-GCGCCCATAACTGTGGTGTT-3' | 1326 | 1.89 |
| Olfml2b | [NM_177068](http://www.ncbi.nlm.nih.gov/nuccore/NM_177068.4) | 5’-ACCTGAGCACGCAGAAGGA-3' | 1952 | 5’-CCCACAGATGACAAAGCAGTTG-3' | 2031 | 1.85 |

Primers were designed, when possible, to span an exon/exon junction to avoid amplification from potentially contaminating genomic DNA. Primer sequences were subjected to BLAT analysis (http://genome.ucsc.edu) to confirm their specificity. The ENSEMBLE genome browser contains the transcript and exon information for the genes investigated in this study (see Accession numbers).
