# Supplementary material for: Brain Transcriptional Responses to High-Fat Diet in Acads-Deficient Mice Reveal Energy Sensing Pathways
Source: PLoS One. 2012 Aug 22;7(8):e41709. doi: 10.1371/journal.pone.0041709 (PMC3425564; doi:10.1371/journal.pone.0041709)
Supplement: Table S3 — Analysis of variance results for acylcarnitines. (DOC) [file pone.0041709.s004.doc]

**Table S3: Two-factor analysis of variance results for plasma acylcarnitines.**

|  | | **Diet** | | **Strain** | | **Diet x Strain** | |
| --- | --- | --- | --- | --- | --- | --- | --- |
| **Symbol** | **Biochemical Name** | ***F*** | ***P-value*** | ***F*** | ***P-value*** | ***F*** | ***P-value*** |
| **C2** | Acetyl-L-carnitine | 5.37 | 0.0324 | 0.08 | 0.7796 | 0.12 | 0.7329 |
| **C3** | Propionyl-L-carnitine | 1.18 | 0.2912 | 0.08 | 0.7808 | 0.14 | 0.7137 |
| **C3-DC/C5-OH** | Malonyl-L-carnitine / Hydroxyvaleryl-L-carnitine | 2.37 | 0.1409 | 0.04 | 0.8424 | 2.7 | 0.1179 |
| **C4** | Butyryl-L-carnitine | 5.4 | 0.0321 | 69.21 | <.0001 | 9.55 | 0.0063 |
| **C4-DC** | Fumaryl-L-carnitine | 0.16 | 0.6982 | 0.18 | 0.6739 | 0.4 | 0.5331 |
| **C4-OH** | Hydroxybutyryl-L-carnitine | 0.64 | 0.4349 | 1 | 0.3314 | 0.34 | 0.5656 |
| **C5** | Valeryl-L-carnitine | 0.93 | 0.3468 | 8.29 | 0.01 | 1.38 | 0.2557 |
| **C5-1** | Glutaconyl-L-carnitine | 6.96 | 0.0167 | 0.4 | 0.5334 | 0.00 | 0.9745 |
| **C5-DC** | Glutary-L-carnitine | 0.88 | 0.3619 | 0.29 | 0.594 | 0.01 | 0.915 |
| **C6** | Hexanoyl-L-carnitine | 3.94 | 0.0627 | 0.03 | 0.8546 | 0.41 | 0.5313 |
| **C8** | Octanoyl-L-carnitine | 1.6 | 0.2217 | 0.28 | 0.6057 | 0.15 | 0.7014 |
| **C8-1** | Octenoyl-L-carnitine | 6.11 | 0.0236 | 1.83 | 0.1926 | 0.98 | 0.3354 |
| **C8-DC** | Octenedioyl carnitine | ND | ND | ND | ND | ND | ND |
| **C10** | Decanoyl-L-carnitine | 0.97 | 0.3382 | 0.71 | 0.411 | 1.31 | 0.2669 |
| **C10-1** | Decenoyl-L-carnitine | 0.32 | 0.5786 | 0.21 | 0.6556 | 2.35 | 0.1426 |
| **C10-2** | Decadienyl-L-carnitine | ND | ND | ND | ND | ND | ND |
| **C10-3** | Decatrienoyl carnitine | 0.01 | 0.9311 | 1.42 | 0.2497 | 0.41 | 0.5321 |
| **C12** | Dodecanoyl-L-carnitine | 22.94 | 0.0001 | 0.45 | 0.5094 | 0.76 | 0.3935 |
| **C12-OH/C10-DC** | Hydroxy-dodecanoyl carnitine or Sebacoyl carnitine | ND | ND | ND | ND | ND | ND |
| **C14** | Tetradecanoyl-L-carnitine | 10.21 | 0.005 | 1.06 | 0.3165 | 0.03 | 0.8706 |
| **C14-1** | Tetradecenoyl-L-carnitine | 0.04 | 0.8428 | 0.04 | 0.8422 | 0.18 | 0.6769 |
| **C14-2** | Tetradecadienyl-L-carnitine | 0.02 | 0.8872 | 0.55 | 0.4676 | 0.02 | 0.8825 |
| **C14-OH/C12-DC** | Hydroxy-tetradecanoyl carnitine or Dodecanedioyl carnitine | 0.47 | 0.5039 | 0.19 | 0.6644 | 1.53 | 0.2324 |
| **C16** | Hexadecanoyl-L-carnitine | 1.51 | 0.2349 | 0.02 | 0.8791 | 0.04 | 0.847 |
| **C16-OH** | Hydroxyhexadecanoyl-L-carnitine | 1.64 | 0.2159 | 2.32 | 0.1453 | 1.61 | 0.2202 |
| **C18** | Octadecanoyl-L-carnitine | 11.36 | 0.0034 | 0.47 | 0.5029 | 1.97 | 0.178 |
| **C18-1** | Octadecenoyl-L-carnitine | 0.02 | 0.8823 | 0.27 | 0.6107 | 0.01 | 0.9379 |
| **C18-2** | Octadecadienyl-L-carnitine | 3.86 | 0.065 | 1.8 | 0.1965 | 1.02 | 0.3252 |
| **C18-OH** | Hydroxyoctadecanoyl-L-carnitine | 3.93 | 0.0628 | 1.84 | 0.1921 | 7.6 | 0.013 |
| **C18-1-OH** | Hydroxyoctadecenoyl-L-carnitine | 0.18 | 0.6798 | 1.46 | 0.2433 | 0.35 | 0.56 |
| **C18-DC/C20-OH** | Hydroxy-eicosanoyl carnitine or Octadecanedioyl carnitine | 0.53 | 0.4766 | 0.01 | 0.9369 | 1.04 | 0.3217 |
| **C20** | Eicosatetranoyl-L-carnitine | 2.7 | 0.1175 | 6.51 | 0.0201 | 0.86 | 0.3659 |
| **C22** | Docosahexaenoyl-L-carnitine | 0.13 | 0.7232 | 0.56 | 0.4647 | 0.08 | 0.7858 |

Main effects of diet (high-fat, low-fat), strain (*Acads-/-*, *Acads+/+*), and their interaction on plasma acylcarnitine levels. *F*, analysis of variance (ANOVA) *F*-test statistic. *P*, probability of obtaining *F*-test statistic. ND, non-detectable level in one or more of the experimental groups.
