# Supplementary material for: Brain Transcriptional Responses to High-Fat Diet in Acads-Deficient Mice Reveal Energy Sensing Pathways
Source: PLoS One. 2012 Aug 22;7(8):e41709. doi: 10.1371/journal.pone.0041709 (PMC3425564; doi:10.1371/journal.pone.0041709)
Supplement: Table S4 — Quantitative real-time RT-PCR validation in whole brain of microarray data for selected genes in the experimental comparison: Acads−/− HF vs. Acads+/+ HF. (DOC) [file pone.0041709.s005.doc]

**Table S4:** Quantitative real-time PCR validation in brain of microarray data for selected genes (*Acads-/-* HF vs. *Acads-/-* LF).

|  |  | **Microarray** | | **Quantitative RT-PCR** | |
| --- | --- | --- | --- | --- | --- |
| **Gene Symbol** | **Gene name** | **Fold change** | ***P*-value** | **Fold change** | ***P*-value** |
|  |  |  |  |  |  |
| **1700030F18Rik** | **RIKEN cDNA 1700030F18 gene** | **-1.77** | **0.0029** | **-1.32** | **0.0258** |
| 5830454E08Rik | RIKEN cDNA 5830454E08 gene | 1.70 | 0.0038 | -1.45 | 0.1089 |
| **Acad10** | **Acyl-Coenzyme A dehydrogenase family, member 10** | **-1.66** | **0.0364** | **1.11** | **0.0336** |
| Acot11 | Acyl-CoA thioesterase 11 | -1.79 | 0.0327 | 1.06 | 0.7981 |
| Aplp2 | Amyloid beta (A4) precursor-like protein 2 | -1.78 | 0.0035 | -1.02 | 0.7859 |
| Arhgap20 | Rho GTPase activating protein 20 | -2.38 | 0.0332 | -1.02 | 0.8935 |
| Atp7a | ATPase, Cu++ transporting, alpha polypeptide | -2.08 | 0.0380 | -1.08 | 0.4158 |
| **AW112010** | **Expressed sequence AW112010** | **1.73** | **0.0163** | **-1.40** | **0.0492** |
| Cask | Calcium/calmodulin-dependent serine protein kinase | -2.18 | 0.0151 | 1.02 | 0.8666 |
| **Clic6** | **Chloride intracellular channel 6** | **-2.96** | **0.0013** | **1.20** | **0.0396** |
| Col4a3bp | Procollagen, type IV, alpha 3 binding protein | -3.10 | 0.0000 | -1.15 | 0.4801 |
| Cox8a | Cytochrome c oxidase, subunit VIIIa | 1.67 | 0.0475 | -1.02 | 0.4886 |
| **Dock5** | **Dedicator of cytokinesis 5** | **-1.64** | **0.0108** | **-1.36** | **0.0379** |
| **F2rl2** | **Coagulation factor II (thrombin) receptor-like 2** | **-2.26** | **0.0036** | **-1.50** | **0.0148** |
| Fggy | FGGY carbohydrate kinase domain containing | -2.48 | 0.0034 | 1.29 | 0.1479 |
| Gm941 | Gene model 941, (NCBI) | -4.30 | 0.0153 | -1.02 | 0.7638 |
| Gpr137c | G protein-coupled receptor 137C | 2.46 | 0.0149 | 1.06 | 0.3020 |
| Gpr44 | G protein-coupled receptor 44 | -1.65 | 0.0447 | -1.60 | 0.0901 |
| **Gpr61** | **G protein-coupled receptor 61** | **-2.93** | **0.0066** | **-1.28** | **0.0343** |
| Insrr | Insulin receptor-related receptor | -3.13 | 0.0284 | 1.11 | 0.5108 |
| Map3k2 | Mitogen-activated protein kinase kinase kinase 2 | -2.15 | 0.0184 | 1.08 | 0.6355 |
| Mapk15 | Mitogen-activated protein kinase 15 | -1.65 | 0.0182 | -1.12 | 0.5421 |
| Mx2 | Myxovirus (influenza virus) resistance 2 | -2.62 | 0.0282 | -1.14 | 0.4370 |
| Ndufc1 | NADH dehydrogenase (ubiquinone) 1, subcomplex unknown, 1 | 2.17 | 0.0374 | 1.06 | 0.1465 |
| **Neurog3** | **Neurogenin 3** | **-1.65** | **0.0210** | **-2.16** | **0.0485** |
| Npc1 | Niemann Pick type C1 | -2.61 | 0.0271 | 1.11 | 0.4742 |
| Nr3c1 | Nuclear receptor subfamily 3, group C, member 1 | -2.53 | 0.0238 | 1.06 | 0.2989 |
| Nrf1 | Nuclear respiratory factor 1 | -4.26 | 0.0443 | 1.08 | 0.3199 |
| Olfml2a | Olfactomedin-like 2A | -1.91 | 0.0378 | -1.05 | 0.8257 |
| Olfml2b | Olfactomedin-like 2B | 1.60 | 0.0290 | -1.24 | 0.2471 |
| **Plekha2** | **Pleckstrin homology domain-containing, family A member 2** | **-2.25** | **0.0307** | **1.22** | **0.0339** |
| **Prlhr** | **Prolactin releasing hormone receptor** | **-1.89** | **0.0480** | **-1.83** | **0.0442** |
| **Ptgfr** | **Prostaglandin F receptor** | **-2.66** | **0.0024** | **-1.85** | **0.0367** |
| Ptprcab | Protein tyrosine phosphatase, receptor type | -1.69 | 0.0138 | -1.95 | 0.0883 |
| Qscn6 | Quiescin Q6 | 1.73 | 0.0354 | 1.18 | 0.3309 |
| **Rassf10** | **Ras association (RalGDS/AF-6) domain family member 10** | **2.96** | **0.0259** | **-1.42** | **0.0312** |
| Rpl11 | Ribosomal protein L11 | 2.17 | 0.0028 | -1.03 | 0.6703 |
| Sacs | Sacsin | -2.98 | 0.0334 | 1.1 | 0.6286 |
| Sass6 | Spindle assembly 6 homolog (C. elegans) | -1.79 | 0.0027 | 1.00 | 0.9673 |
| Scn2b | Sodium channel, voltage-gated, type II, beta | 1.82 | 0.0068 | -1.09 | 0.8104 |
| **Slc22a13** | **Solute carrier family 22 (organic cation transporter), member 13** | **-1.83** | **0.0274** | **-1.83** | **0.0249** |
| Tnfsf18 | Tumor necrosis factor (ligand) superfamily, member 18 | -2.45 | 0.0205 | 1.14 | 0.7790 |
| Ugt2b36 | UDP glucuronosyltransferase 2 family, polypeptide B36 | 3.84 | 0.0148 | -1.19 | 0.7434 |

Positive fold change indicates increased expression, negative value indicates decreased expression in the first strain of the comparison.

Genes were selected based on a significance level of *P* < 0.05 and a 1.6-fold or greater change in expression, i.e., ratio of the normalized signal intensity.
