# Supplementary material for: Brain Transcriptional Responses to High-Fat Diet in Acads-Deficient Mice Reveal Energy Sensing Pathways
Source: PLoS One. 2012 Aug 22;7(8):e41709. doi: 10.1371/journal.pone.0041709 (PMC3425564; doi:10.1371/journal.pone.0041709)
Supplement: Text S1 — Quantitative real-time RT-PCR validation of microarray data for selected genes. (DOC) [file pone.0041709.s006.doc]

**Text S1:** **Quantitative real-time RT-PCR validation of microarray data for selected genes.**

To validate transcripts, we initially selected a single comparison, i.e., J HF v J LF (Table S4). Only 13 out of 43 genes selected (30%) from the microarray results were confirmed, and of these 5 were changed in opposite direction. Eight (8) genes showed a comparable fold-change in the same direction in both methods: *Dock4*, *F2rl2*, *Gpr61*, *Neurog3*, *Prlhr*, *Ptgfr*, *Slc22a13*, and *1700030F18Rik*. Two possible reasons for the low confirmation rate are: 1) low signal intensity, i.e., more than 50% of the genes selected for validation had a signal intensity of <2000, and/or 2) high coefficient of variation for differential expression, i.e., the calculated coefficient of variation (CV) for differentially expressed genes was 4–61% across the entire signal range. It is also possible that high cellular heterogeneity in brain may have reduced the ability to detect molecular phenotypes (Karsten et al., 2008).

Karsten SL, Kudo LC, Geschwind DH (2008) Gene expression analysis of neural cells and tissues using DNA microarrays. Curr Protoc Neurosci Chapter 4: Unit 4 28.
